# Comparison of Experimental and Calculated Ion Mobilities of Small Molecules in Air

**DOI:** 10.1155/2016/6246415

**Published:** 2016-05-19

**Authors:** Frank Gunzer

**Affiliations:** Information Engineering and Technology Faculty, German University in Cairo, El Tagamoa El Khames, Cairo, Egypt

## Abstract

Ion mobility spectrometry is a well-known technique for analyzing gases. Examples are military applications, but also safety related applications, for example, for protection of employees in industries working with hazardous gases. In the last 15 years, this technique has been further developed as a tool for structural analysis, for example, in pharmaceutical applications. In particular, the collision cross section, which is related to the mobility, is of interest here. With help of theoretic principles, it is possible to develop molecular models that can be verified by the comparison of their calculated cross sections with experimental data. In this paper, it is analyzed how well the ion trajectory method is suitable to reproduce the measured ion mobility of small organic molecules such as the water clusters forming the positively charged reactant ions, simple aromatic substances, and n-alkanes.

## 1. Introduction

Ion mobility spectrometry (IMS) has been a tool for the analysis of gases for a long time. Famous for their small device size in the centimeter range, their fast response times in the ms range, and their very high sensitivity in the lower ppbv range, IMS detectors can be found in a number of quite different locations and applications [1]. Much younger is the scientific application of IMS in order to characterize the structure of analytes. Already in 2004 a review [2] stated that IMS has matured enough to become a mainstream research methodology for structural analysis, especially in combination with mass spectrometry. Applications in gas phase peptide ion structure analysis can be found, for example, in [3], where IMS measurements combined with molecular modeling allowed for the determination of folding structures and backbone orientations. An overview over the variety of structure analysis based on IMS can be found in, for example, [4]. A detailed description of the different mathematical approaches employed in order to calculate the most important parameter for structural information, the collision cross section (which furthermore determines the ions' mobility), can be found in [5].

Basically, there are three methods available to calculate the collision cross section from model structures and thus the ion mobility [6–8]. They represent different compromises between accuracy and calculation time. The method known for delivering the best results and which is also the mathematically most demanding (and thus leading to the longest calculation times) is the Trajectory Method. In this method, the angle between the incoming trajectory and outgoing trajectory of a particle colliding with the analyte is used to determine the collision cross section [9]:

(1) ΩTM=18π2∫02πdθ∫0πdϕsin⁡ϕ∫02πdγπ8μkBT3·∫0∞dge−μg2/2kBTg5∫0∞db2b1−cos⁡χθ,ϕ,γ,b.

The variables *θ*, *ϕ*, and *γ* are the Euler-Angles for the collision geometry between analyte and drift gas, *χ*(*θ*, *ϕ*, *γ*, *b*) is the scattering angle, *g* is the relative velocity of the drift gas, *b* is the impact parameter defining how far the drift gas atom is positioned from the *x*-axis, *μ* is the reduced mass, *k*_*B*_ is the Boltzmann Constant, and *T* is temperature in Kelvin. The collisions are assumed to be elastic. The standard approach is that model structures are optimized (typically with help of quantum-chemical calculations), and then their collision cross section is calculated, from which finally the ion mobility can be calculated [9, 10]:

(2) $$\begin{array}{c} K=\frac{3}{16}\frac{q}{N}{\left(\;\frac{1}{M}+\frac{1}{m}\;\right)}^{0.5}{\left(\;\frac{2\pi }{k_{B}T}\;\right)}^{0.5}\frac{1}{\Omega_{TM}}. \end{array}$$

*M* and *m* are the masses of the (neutral) drift gas and the analyte, *q* is the analyte's charge, and *N* is the number density of the drift gas. This mobility value is directly available from flight time measurements in an ion mobility spectrometer [1]:

(3) $$\begin{array}{c} K=\frac{L^{2}}{t_{D}U}. \end{array}$$

Here *L* is the length of the drift tube, *U* is the voltage drop over the whole drift tube length, and *t*_*D*_ is the analyte's drift time directly obtainable in the measurements.

To calculate the (optimized) geometry, a lot of options exist regarding the mathematical approach. For smaller molecules, density functional theory (DFT) is an often used example. For larger molecules as in biological or pharmaceutical applications, DFT takes too long, and thus other approaches such as force field calculations and/or molecular modeling are used. For smaller molecules, on which the focus lies in this paper, DFT allows choosing from a variety of functionals, which can be combined with a variety of basis sets, in order to optimize the structure. All these combinations represent again different compromises between accuracy and calculation speed. From DFT calculations, the structural parameters of the analyte molecule can be obtained. The calculation of the collision cross section requires further information in the form of the partial charge distribution over the analyte molecule's atoms. The most well-known method to calculate this distribution is the Mulliken approach [11], which basically distributes partial charges according to how many atom orbital functions contribute to molecular orbital representations. This information is mathematically directly obtained during the structure optimizations, and thus the Mulliken charges are easy and fast to calculate. However, their magnitude is sometimes unrealistic, and much more important, their magnitude depends strongly on the before mentioned basis set and its size. Different calculations thus typically yield different Mulliken charges. Correspondingly, further approaches have been developed to avoid this dependence on the basis set. What is problematic for all these approaches is that there is no clear definition where an atom begins and where it ends in a molecule, so it is difficult to assign partial charges to the atoms. Another also well-known method is the Merz-Kollman-Singh (MK, [12]) scheme. This scheme belongs to a family of techniques where the reproduction of the electrostatic potential around an atom by assigning partial charges is the goal. The different schemes in this family differ by the way they fit the charges to the potential, that is, in the number and distribution of fit points. A third, similarly well-known technique is the Hirshfeld population analysis [13]. Here the atoms' partial charges are calculated by assuming that the electron density in a certain point of the molecule's space is shared among the surrounding atoms. Each atom contributes in a magnitude proportional to the free-electron density and distance to the nuclei. These different approaches normally yield completely different numerical values for the partial charges (but always summing up to the total charge of the molecule). In recent studies, there have been different results regarding which partial charge scheme is better suited, for example, when compared with experimental values. When different basis sets were used for the calculation of the molecular structures, Hirshfeld behaved quite stably, while Mulliken behaved unstably and showed a large variation of the computed charges when increasing the basis set size. Hirshfeld on the other hand showed often significantly different values [14]. When calculating, for example, charge transfers in organic dyes, Mulliken and MK showed the least variations, while Hirshfeld showed inconsistent results; for larger molecules, Mulliken provided poor results [15]. Another more recent example is the calculation of dipole moments of Diheteroaryl Ketones and Thioketones [16]. There MK was the best approach, followed by Mulliken, and Hirshfeld was the worst; in contrast to this, when reproducing tendencies for a larger group of conformers, Hirshfeld showed better results, followed by MK and Mulliken.

In this paper, we have analyzed how well the Trajectory Method can reproduce the experimental ion mobilities that we measured for small molecules. Thus, we have calculated minimum structures with the help of DFT and different partial charge methods and calculated with this data the ion mobility using an application called MOBCAL in a version which has been improved for calculations with N_2_ as drift gas [17]. After a short description of the experimental and computational procedures, the paper will show direct comparisons of the calculated and the measured mobilities for analytes of different sizes.

## 2. Experimental

The experimental mobilities have been obtained with a commercial IMS spectrometer which has been described thoroughly in previous publications [18] so that here only a short summary is given. The IMS device has three distinct sections: an ionization region, a drift region, and a collector region. The ionization region has a length of 0.5 cm; the drift region's length is 5.5 cm (diameter 2 cm), followed by the collector region of 2 mm length with the detector, a Faraday Cup, located at its end. In the ionization region, positively charged reactant ions are created in ambient air with the help of free electrons emitted by an electron gun. These reactant ions then ionize the analyte. The analyte ions are injected from the ionization region into the drift region by an electric field of strength 1000 V/cm. The field strength in the drift region is ca. 350 V/cm. In the drift region, the ions fly against a drift gas flow of 200 mL/min. The drift times are in this set-up ca. 6 ms and longer. The humidity level is kept relatively low (water concentration at 292 K below 200 ppbv, dew point of 181.1 K).

The quantum-chemical calculations have been performed using the Gaussian software suite (version 9 D.01 [19]). The B3LYP functional together with the 6-311+G(d) basis set has been used for the structure optimizations and the partial charge calculations according to Mulliken, MK, and Hirshfeld. Although B3LYP is only a general purpose functional with limited precision, we assume that the errors introduced by the Trajectory Method are larger and thus did not choose a more refined functional. A similar argumentation led to the choice of the basis set, which, however, is considered to be large enough in order to get a good estimation for the charges.

The program used for the calculation of the collision cross section and the theoretic ion mobilities is MOBCAL in the extended version for N_2_ drift gas and heavier atoms. Although our measurements were using ambient dry air as drift gas, we consider the calculated mobilities suitable for such a comparison since, according to the generally valid Blanc's Law of Ion Mobilities [20, 21], the difference between mobilities in air and in nitrogen should be small. With help of the Langevin polarization limit expression, the difference between measurements in oxygen and nitrogen can be calculated as [22]

(4) $$\begin{array}{c} K_{O_{2}}=K_{N_{2}}\sqrt{\frac{\left(m+M_{O_{2}}\right)/m\left(D_{O_{2}}-1\right)}{\left(m+M_{N_{2}}\right)/m\left(D_{N_{2}}-1\right)}}, \end{array}$$

where *K*_O_2_/N_2__ are the mobility constants in the corresponding medium, *D*_O_2_/N_2__ and *M*_O_2_/N_2__ are the dielectric constants and the molecular masses of the corresponding medium, and *m* is the molecular mass of the analyte. Thus, the corrections between mobility in oxygen and nitrogen are small. According to Blanc's Law of Ion Mobilities these have to be weighted by the molar composition of air with respect to these two gases in order to get the mobility in air, leading to a total difference of mobilities in nitrogen and air of a few percent (but depending on the analyte's mass). Experimental studies showed indeed variations of less than 3%, for example, in [23].

The analyte substances have been acquired from Sigma Aldrich (http://www.sigmaaldrich.com/) with a purity of at least 95%. They have been directly used without any further processing or purification.

## 3. Results and Discussion

The first group of molecules to be discussed is the water clusters that form the analyte ionizing reactant ions in IMS [1]. These are of the form (H_3_O^+^)(H_2_O)_*n*_ with *n* depending on the temperature and the humidity level in the device. Typical values are zero to five; under standard conditions, a significant number of molecules can be found for *n* between three and five. The IMS peak obtained from these reactant ions (called RIP, [1]) is thus a mixture of different ions that cannot be separated with IMS alone. We thus compare our MOBCAL calculations with values obtained by another theoretical approach that could successfully calculate with high confidence the flight time distribution measured in IMS experiments. In that approach, particle tracing together with statistical diffusion simulation and a Monte Carlo based reaction simulation (involving the concentration of the reaction partners, here the water clusters of different size, and their reaction constants) has been used to simulate RIP spectra [24].  Table 1 shows the corresponding comparison. For smaller clusters, the calculated mobilities are smaller than those calculated by [24]; for larger clusters, they are actually too large. All partial charge approaches show the same behavior. Except for one case, the value calculated with Mulliken partial charges is in between those calculated with the other two approaches, the variations being below ten percent. The mean unsigned error (MUE) when compared with the values calculated in [24] is for all three approaches around 11%; however, for the largest clusters, the error is as large as 20%. The MK approach shows here values with the smallest error, and also the maximum error is less than that for the other partial charge methods. In particular, for the larger clusters the difference approaches 10%, so for this case the MK method clearly outperforms the other two. In summary, however, it seems that only for larger clusters (*n* = 4, mass 91 dalton) the Trajectory Method seems to be able to calculate values close to the ones successfully used in [24] to reproduce experimental values.

**Figure 1:**
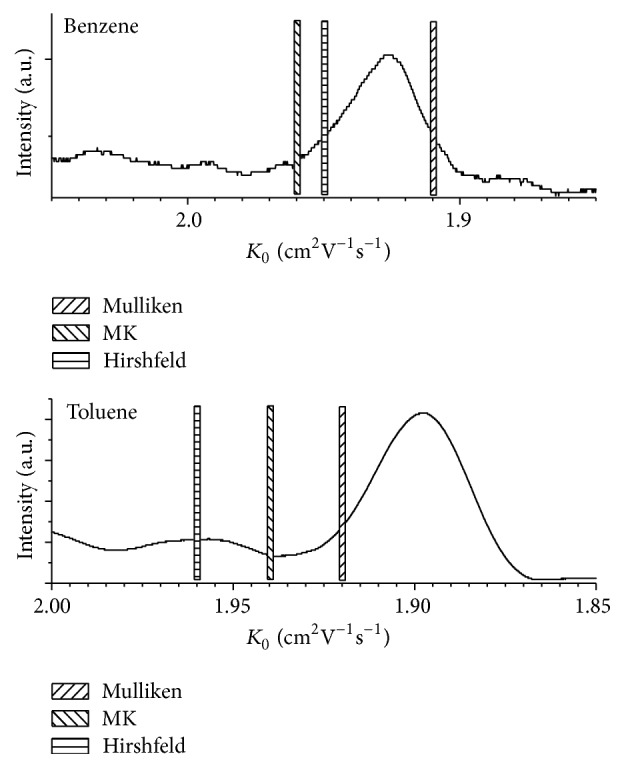
Measured IMS spectrum for benzene and toluene (solid line) and the calculated mobilities when the analyte is clustered with one water molecule (bars).

The second group contains small aromatic systems, here benzene and toluene. For these, an interesting problem appeared. Both substances were investigated by our group in the past, and under those conditions it appeared that the ionization mechanism is not the typical protonation, but electron abstraction. This is more common when using as low humidity levels as we have in our set-up [26]. For such structures, the calculated mobilities yielded too high values. For benzene we measured a mobility of 1.94 cm^2^V^−1^s^−1^ (peak center; the peak width determines the device's resolving power, which has in our set-up a value of ca. 40 which is a standard value for commercial devices). The calculated value, however, was 2.20 cm^2^V^−1^s^−1^ for MK charges (2.22 cm^2^V^−1^s^−1^ for Hirshfeld charges) and thus showed a large error. However, it is known in IMS that the analytes form clusters with H_2_O after ionization [1], the degree again depending on temperature and humidity level. Thus, we calculated mobilities of (benzene)^+^(H_2_O) and (toluene)^+^(H_2_O).  Figure 1 shows the corresponding IMS spectra together with the calculated values represented as bars.

When using the aromatic compound clustered with water, the calculated values are much closer to the measured values. The differences are below 5% in all cases. The interesting question is if this can be seen as a clear indication that the measured signal is indeed caused by the analyte clustered with water. One common way to measure this experimentally is the hyphenation of IMS with mass spectrometry (MS); but since IMS work at atmospheric pressure and mass spectrometers at vacuum, an interface has to be used in order to separate these two pressure areas. This normally means an opening with very small diameter in the 100 s of micrometer range, where additional clustering of all molecules in the gas stream can be observed, but also declustering. The masses obtained by IMS-MS do not necessarily represent the substances in the IMS; in the case of clustering, further calculations have to show if theoretic mobility calculations can help here. Regarding the partial charges, the Mulliken charges yield for both benzene and toluene the best result. For benzene the calculated mobility is too low, but for toluene it is too high. The Hirshfeld charges are better for benzene where the error is comparable to that of the Mulliken charges but with opposite sign, but much worse in the case of toluene where the MK charges are better. Here the trend that with increased analyte mass the calculated mobilities are closer to the experimental ones could not be observed.

**Figure 2:**
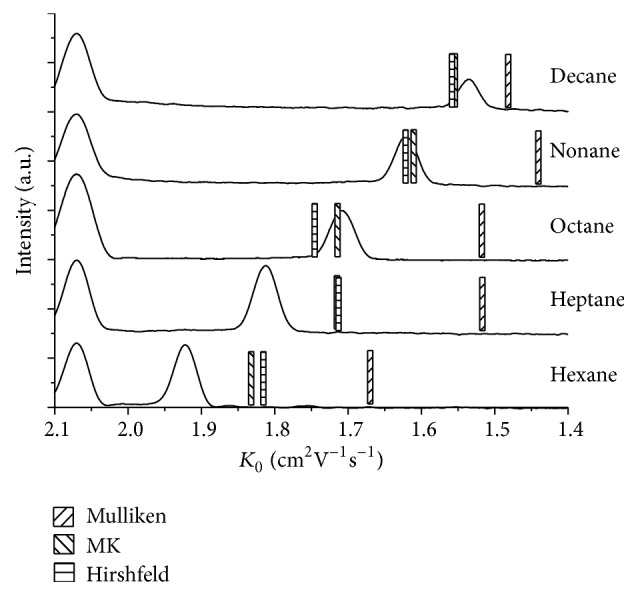
Experimental IMS spectra for n-alkanes (solid lines) and the calculated mobilities (bars). The peak at 2.07 cm^2^V^−1^s^−1^ is created by the reactant ions and thus present in all alkane spectra.

The third group to be presented here is n-alkanes ranging from n-hexane with a mass comparable to benzene to n-decane which has a mass comparable to pharmaceutically interesting analytes such as Acetaminophen. These alkanes have the problem that, due to their charge symmetry, the ionization via protonation is not possible (no attachment point for the proton). It has been found out in the past that electron abstraction leads to ionization in IMS (for details, see [25]). Here we have again investigated clusters formed with one water molecule, which has been attached to the center of each alkane.  Figure 2 shows the corresponding IMS spectra together with the calculated values.

When using the Mulliken partial charge approach, the calculated mobilities are all too low. While the experimental values show a tendency towards lower mobility for higher chain length, the mobilities calculated using Mulliken charges do not show that tendency (n-octane and n-heptane have the same mobility; n-decane has a lower mobility than n-octane but higher than n-nonane). The mean unsigned error is again ca. 11%.

When using the MK partial charges or the Hirshfeld partial charges, the deviations are much less. Both show an increase in mobility from n-heptane to n-octane which is experimentally not observed (Mulliken charges showed here identical mobilities), but apart from that point the experimental mobilities are quite well reproduced by the calculations. In two cases, both partial charge approaches actually yield the same mobility; in the case of n-nonane, the difference is less than one percent. The error is in general less for longer chains, and the error for MK charges is slightly smaller than that for Hirshfeld charges. The mean unsigned error is 2.6% for MK and 3.0% for Hirshfeld charges.  Table 2 summarizes these findings.

The maximum MUE in these simulations was smaller than 12%. The average error is much less, especially for the MK and Hirshfeld charges, where it is near 5%. This error is in the same range as errors published, for example, in a study of biomolecules with much larger mass. In [27], where molecules with masses ranging from 122 dalton to 603 dalton have been investigated, all deviations are within 5% of the experimental values. Smaller molecules such as the ones used here are much more critical. What becomes interesting is the influence of the partial charges. While for the water clusters Mulliken charges yield comparable errors, they are much worse in the case of n-alkanes. MK and Hirshfeld perform in a similar manner, with MK charges yielding slightly lower errors in all cases. From this study, it can be concluded that, for smaller molecules with a maximum mass of 160 dalton, the MOBCAL extended version is capable or reproducing experimental values with errors not much different from those known from other studies. The error is in the range of below 10% when using advanced partial charge calculations like MK or Hirshfeld but can be much higher when using Mulliken partial charges.

## 4. Conclusions

In this paper, it was shown how well calculated ion mobilities compare with experimental values in the case of benzene, toluene, and n-alkanes. Under the assumption that these substances cluster with one water molecule, the calculated mobilities (for B3LYP//6-311G+(d) optimized structures) are close to the experimental values (difference below 5%) when using MK or Hirshfeld partial charges. Mulliken charges show a much greater error of around 10%. These error values are comparable to those obtained for larger molecules investigated in other studies. Further investigations have to show how well this comparison can be extended to other molecule types typically found in IMS. While the structures shown here are all investigated in the positive mode (where positively charged analyte ions are formed by protonation or electron abstraction), it would be interesting to see how compounds investigated in the negative mode (formed by proton abstraction or electron capture) behave regarding their calculated mobilities. Similarly, the often observed dimer formation in both modes has not been investigated. But based on the data available so far it becomes already clear that the available approaches allow in many cases (here additionally shown for small molecules below 160 dalton) calculating mobilities from model structure with good confidence and thus supporting structural elucidation of unknown substances.

## Figures and Tables

**Table 1:** Calculated ion mobilities of water clusters (H_3_O^+^)(H_2_O)_*n*_ typically found in the RIP (all in cm^2^V^−1^s^−1^). The mean unsigned error (MUE) is calculated in relation to the values obtained by [24].

*n*	[24]	Mulliken	MK	Hirshfeld
0	3.57	3.16	3.00	3.23
1	2.76	2.71	2.65	2.75
2	2.35	2.50	2.45	2.55
3	1.97	2.38	2.26	2.37
4	1.88	2.18	2.13	2.25
MUE (%)		11.29	10.45	11.68

**Table 2:** Calculated ion mobilities of n-alkanes (clustered with one water molecule) in comparison with experimental values (all in cm^2^V^−1^s^−1^). Experimental values taken from [25].

Substance	Exp.	Mulliken	MK	Hirshfeld
Hexane	1.92	1.67	1.84	1.83
Heptane	1.81	1.52	1.72	1.72
Octane	1.71	1.52	1.73	1.75
Nonane	1.62	1.44	1.63	1.64
Decane	1.53	1.48	1.56	1.56
MUE (%)		10.9	2.5	3.0
